# Seasonal Variation of Plaque Psoriasis in Relation to Individualized MED-Adjusted Ultraviolet Exposure: A Cross-Sectional Study in Poland

**DOI:** 10.3390/jcm15072708

**Published:** 2026-04-03

**Authors:** Michał Niedźwiedź, Agnieszka Czerwińska, Janusz Krzyścin, Joanna Narbutt, Aleksandra Lesiak

**Affiliations:** 1 Department of Dermatology, Paediatric Dermatology and Oncology, Medical University of Lodz, 90-419 Lodz, Poland; joanna.narbutt@umed.lodz.pl (J.N.); aleksandra.lesiak@umed.lodz.pl (A.L.); 2 International Doctoral School, Medical University of Lodz, 90-419 Lodz, Poland; 3 Institute of Geophysics, Polish Academy of Sciences, 01-452 Warsaw, Poland; aczerwinska@igf.edu.pl (A.C.); jkrzys@igf.edu.pl (J.K.); 4 Laboratory of Autoinflammatory, Genetic and Rare Skin Disorders, Medical University of Lodz, 92-215 Lodz, Poland

**Keywords:** plaque psoriasis, seasonality, ultraviolet radiation, minimal erythema dose, exposure–response, non-linear models, environmental exposure, patient-reported outcomes, geoepidemiology, mixed-effects modeling

## Abstract

**Background:** Patient-perceived seasonality of psoriasis is frequently reported, yet the independent contribution of objectively quantified, individualized ultraviolet (UV) exposure remains insufficiently characterized. We evaluated seasonal variation in plaque psoriasis and its association with geocoded, phototype-adjusted ambient antipsoriatic radiant exposures (ARE) using mixed-effects modeling. **Methods:** This cross-sectional study included 119 adults with plaque psoriasis (476 seasonal observations). Participants rated seasonal disease courses using a 7-point scale. Ambient ARE was geocoded to residential postal codes and quantified as a behaviorally weighted dose normalized to individual minimal erythema dose (MED). Mixed-effects logistic regression models, adjusted for relevant confounders, estimated associations with seasonal improvement and worsening. **Results:** Seasonality was reported by 89.9% of participants (*p* < 0.001). Summer was the most favorable season, whereas winter was the most detrimental. The highest ARE quartile was independently associated with increased odds of improvement (OR 4.65, 95% CI 2.04–10.58, *p* < 0.001) and reduced odds of worsening (OR 0.16, 95% CI 0.08–0.33, *p* < 0.001). Crucially, continuous quadratic modeling revealed a significant inverted U-shaped relationship between UV exposure and improvement, with an estimated turning point of 3.85 (95% CI 1.88–5.82, *p* < 0.001) for the declared daily ARE (UV_decl_) normalized by MED. Beyond this threshold, the probability of improvement attenuated. The protective effect against seasonal worsening remained linear. **Conclusions:** Psoriasis seasonality demonstrates a robust exposure–response association relationship with ambient UV. The estimated turning point (UV_decl_/MED = 3.85) within our modeled exposure metric is exploratory and hypothesis-generating. It suggests an association where moderate UV exposure correlates with patient-perceived benefits, but these diminish at very high levels. This threshold requires external prospective validation before being considered a clinically actionable recommendation.

## 1. Introduction

Psoriasis is a chronic, immune-mediated inflammatory skin disease affecting approximately 2–3% of the adult population in Europe and representing a substantial clinical and psychosocial burden [1,2]. Seasonal fluctuation of disease activity has been described for decades, with early reports consistently noting winter exacerbation and summer improvement in a considerable proportion of patients [3,4,5,6]. Contemporary survey-based studies suggest that 45–75% of patients perceive some degree of seasonality, although the direction and magnitude of change differ across regions and climates [6,7,8]. Population-level analyses of healthcare utilization and internet search trends further demonstrate reproducible winter peaks and summer troughs in psoriasis-related activity in both hemispheres [9,10,11]. Ultraviolet (UV) radiation is considered a principal biological mediator of seasonal variation due to its immunomodulatory effects, including suppression of Th17-driven inflammation and induction of keratinocyte apoptosis [12,13,14].

However, large administrative database studies have yielded inconsistent findings, with some reporting minimal or absent seasonal patterns in diagnosis rates or histopathological confirmation [15,16]. Such discrepancies likely reflect methodological heterogeneity, including differences in outcome definitions, latitude, behavioral exposure, and reliance on aggregate rather than patient-level data [6,11].

Most prior studies have assessed seasonality indirectly through clinical visits, hospitalizations, or search activity rather than integrating subjective disease perception with objectively quantified environmental exposure [7,9,11]. Environmental factors beyond UV radiation, such as humidity, air pollution, and physical activity, have also been implicated in psoriasis modulation but are rarely evaluated simultaneously within multivariable models [9,17,18]. Individual susceptibility characteristics including skin phototype, UV reactivity, age, and occupational circadian disruption may further modify the impact of environmental exposure on disease expression [12,14,18]. Additionally, emerging data suggest that season of treatment initiation may influence therapeutic response, underscoring the relevance of environmental context in psoriasis management [19].

Most prior studies lack individualized UV dosing anchored to constitutional phototype and daily behavior at the patient level. Furthermore, when evaluating environmental impact, it is crucial to conceptually distinguish ‘ambient UV availability’ from the ‘actual skin (personal) antipsoriatic radiant exposure (ARE)’. The latter one is heavily modified by individual outdoor time, clothing habits, and constitutional UV sensitivity (Minimal Erythema Dose). By failing to integrate these behavioral and biological variables, previous models may have obscured the true exposure–response relationships.

Therefore, the aim of the present study was to (1) quantify perceived seasonal variation in psoriasis using a standardized 7-point scale, (2) evaluate patient-reported environmental triggers, (3) estimate residential and individualized ambient ARE based on geocoded data, and (4) identify independent predictors of clinically meaningful seasonal improvement and worsening using multivariable mixed-effects logistic regression models.

## 2. Materials and Methods

This cross-sectional epidemiological investigation systematically examined patient-perceived seasonality in psoriasis vulgaris course and its relationship to environmental exposures among Polish adults. Conducted between June and 15 December 2025, the study employed an online survey methodology targeting patients through three primary recruitment channels: (1) national psoriasis patient organizations (Polskie Towarzystwo Łuszczycy), (2) dermatology clinic electronic waiting lists from 2 healthcare centers in Poland, and (3) targeted advertising within closed Facebook patient support groups.

Inclusion criteria comprised: confirmed physician diagnosis of plaque psoriasis (ICD-10 L40.0), age ≥ 18 years, Polish residency with valid postal code, and ability to complete the Polish-language questionnaire. Exclusion criteria included other primary dermatological diagnoses, inability to provide informed consent, and incomplete responses to core seasonality assessments (≥1 missing seasonal items and/or lack of providing postal code).

The study protocol received institutional review board approval from the Bioethics Committee at the Medical University of Łódź (approval number RNN/204/25/KE, dated 10 June 2025) and adhered strictly to the principles of the Declaration of Helsinki (2013 revision). All participants provided electronic informed consent through a dedicated survey portal prior to accessing questionnaire content. Data was anonymized at collection; no personal identifiers were retained beyond postal code for geocoding. Age, biological sex, height/weight (calculated BMI), postal code (for UV geocoding), shift work pattern (none/night shifts/rotating schedules). Comprehensive comorbidity checklist covering cardiovascular (hypertension, ischemic heart disease), metabolic (diabetes), psychiatric disorders, and psoriasis duration/phenotype. Fitzpatrick phototype was self-reported using a structured instrument emphasizing individual sunburn and tanning response.

Lifestyle and behavioral factors were assessed with Physical activity frequency 7-point Likert scale (7-point ordinal: never → daily), vitamin D supplementation status/dosage (IU/day), and tobacco use frequency (7-point scale). Seasonal disease course was assessed as the primary outcome using a 7-point bidirectional Likert scale applied separately to each season. The scale ranged from 1, indicating significant exacerbation of symptoms, to 7, indicating significant improvement, with a score of 4 representing no perceived change. While this specific 7-point scale has not been formally validated against objective clinical severity measures, such as Psoriasis Area and Severity Index (PASI), Physician Global Assessment (PGA), or Body Surface Area (BSA) in prior psoriasis research, it was explicitly chosen to capture the patient-perceived direction and magnitude of seasonal changes rather than absolute clinical severity. This distinction ensures the outcomes reflect subjective disease burden and prevents the overinterpretation of ratings as strict clinical endpoints. Intermediate values reflected grade worsening or improvement (mild or moderate). A composite primary outcome termed “any seasonality” was defined as the presence of at least one season rated with a score different from 4, indicating any perceived seasonal variation in disease severity.

Perceived environmental triggers were evaluated using the same 7-point bidirectional Likert scale. Participants assessed the perceived impact of opposing environmental conditions, including high versus low sunlight exposure, high versus low humidity, high versus low air pollution, and high versus low levels of physical activity. For each factor, lower scores reflected symptom exacerbation under one condition, whereas higher scores indicated symptom improvement under the opposite condition, with a neutral score of 4 indicating no perceived effect. Improvement was defined as seasonal Likert score 5–7, worsening as 1–3; scores of 4 were considered neutral and included in the non-event category for respective models.

Because measuring the exact actual skin UV dose is not feasible in survey-based epidemiological studies, our research utilizes geospatial and behavioral weighting to model individualized ‘ambient UV availability’. This calculated metric acts as a standardized proxy to estimate the individualized exposure–response relationship. Ultraviolet (UV) exposure was operationalized using four complementary season-specific metrics, constructed to distinguish between objective environmental irradiance, self-declared behavioral exposure, and phototype-adjusted biological dose. For each season, the following indices were calculated:

- Ambient UV—total ARE (J_psor_/m^2^) accumulated between 06:00 and 18:00, calculated from a model of UV distribution over Poland derived from various satellite observations (for more details see Appendix A) and assigned to geocoded residential locations.
- Declared daily ARE (behavioral exposure)—ambient ARE weighted by self-reported average daily time spent outdoors in each season.
- Ambient UV normalized to MED—total (6:00–18:00) ambient ARE divided by individual minimal erythema dose (MED), derived from self-declared phototype, reflecting estimated biologically effective UV dose.
- Declared daily ARE normalized to MED (declared UV/MED)—behaviorally weighted UV exposure further normalized by phototype-specific MED, representing individualized, behavior-adjusted biologically effective UV exposure.

Minimal erythema dose (MED) values were assigned according to standard Fitzpatrick phototype reference ranges, i.e., 200, 250, 300, 450, and 600 J_eryt_/m^2^ for phototypes I to V, respectively [20,21,22].

All statistical analyses were conducted using Stata 19 MP Parallel Edition (StataCorp LLC, College Station, TX, USA). To evaluate the independent association between ultraviolet (UV) exposure and seasonal psoriasis dynamics, multivariable mixed-effects logistic regression models were fitted. All four seasonal observations per participant were included in the analysis. Random intercepts for participant ID were incorporated into the models to account for within-subject clustering across seasons. This approach is essential in repeated-measures analysis to control for the non-independence of data when multiple observations (i.e., four seasonal ratings) are derived from the same individual [23,24].

Declared daily ARE was operationalized using the predefined metric of MED-adjusted declared exposure (UV_decl_/MED). It was analyzed both as a categorical variable (quartiles) and as a continuous variable. Furthermore, a continuous quadratic term was introduced to assess potential non-linear exposure–response association effects. All regression models were adjusted for relevant confounders, including physical activity, vitamin D supplementation, body mass index (BMI), and lesion localization (exposed vs. covered). A *p*-value of <0.05 was considered statistically significant.

## 3. Results

### 3.1. Study Population Characteristics

A total of 119 patients with diagnosed plaque psoriasis were included (response rate 95,7% among complete responses). Mean age was 43.5 ± 12.5 years (range 18–71). Females comprised 68.9% (*n* = 82) (Table 1).

Comorbidities were prevalent (52.1%, *n* = 62), most commonly metabolic disorders. Shift work exposure was reported by 24.4% (*n* = 29). Physical activity was common (81.5%, *n* = 97), though most spent <4 h outdoors daily (76.5%, *n* = 91).

Any form of physical activity was common among the study population, reported by 81.5% (*n* = 97) of participants. While 18.5% (*n* = 22) reported no regular exercise, the active majority followed diverse patterns ranging from occasional to daily routines. Despite this generally active profile, 76.5% (n = 91) of the total cohort spent less than 4 h outdoors daily, indicating that much of this activity may occur in indoor or shaded environments.

**Figure 1:**
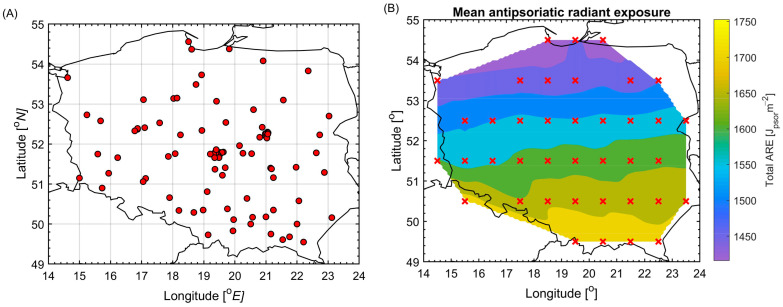
Spatial characterization of ambient UV exposure. (**A**) Geographic distribution of study participants across Poland (red dots). (**B**) Characterization of total ambient antipsoriatic radiant exposure (ARE) in J_psor_/m^2^ accumulated between 06:00 and 18:00 from April to September, derived from a model of UV distribution over Poland based on various satellite observations (see Appendix A) and calculated at the center of grid cells corresponding to patients’ residential locations (red crosses).

Skin phototypes were predominantly fair (*n* = 72; 60.5% of the cohort, including self-reported types I, II, and III). Ambient annual ARE (from April to September) was 1607 ± 73 J_psor_/m^2^ at residence. The corresponding declared ARE after declared phototype adjustment was 494 ± 440 J_psor_/m^2^ (range 0–1696), reflecting substantial inter-individual variability (Figure 1).

### 3.2. Primary Outcome: Seasonality Prevalence

A total of 89.9% of patients (107/119, 95% CI 84.5–95.3%) reported perceiving seasonality in their psoriasis course (defined as ≥1 season rated ≠4 ‘no change’).

### 3.3. Seasonal Patterns

Detailed seasonal distributions revealed clear, non-random patterns (all Wilcoxon signed-rank tests vs. score = 4, *p* ≤ 0.197) (Table 2). Spring was characterized by a balanced, near-neutral course, with 37.8% of patients reporting worsening versus 29.4% reporting improvement (median = 4, *z* = −1.29, *p* = 0.197). Summer was identified as the most favorable season, with 55.5% of patients reporting clinical improvement (scores 5–7) and only 15.9% reporting worsening (scores 1–3) (median = 5, *z* = 4.78, *p* < 0.001). Notably, 39.0% of respondents reported moderate-to-marked improvement. In contrast, autumn showed a net clinical worsening, with 62.8% of participants rating their course between 1 and 3 (median = 3, *z* = −6.43, *p* < 0.001). Winter was the most detrimental season, as 71.4% of patients experienced worsening and only 9.2% noted improvement (median = 2, *z* = −7.12, *p* < 0.001). Specifically, over half of the cohort (51.3%) reported moderate-to-marked winter worsening, corresponding to scores of 1 or 2 on the Likert scale.

Paired comparisons confirmed a pronounced within-individual asymmetry in seasonal deterioration, with worsening reported far more frequently in winter than in summer (McNemar test: *p* < 0.001; OR ≈ 10.4).

### 3.4. Environmental Trigger Perceptions

Scores were assessed using a 7-point bidirectional Likert scale (1 = strong worsening, 4 = no change, 7 = strong improvement). *p*-values were calculated using the Wilcoxon signed-rank test versus the neutral score (4). Environmental factors showed consistent, non-neutral perceptions (all Wilcoxon tests vs. score = 4) (Table 3).

High sunlight exposure emerged as most beneficial (58.8% improvement ratings) and low sunlight as most detrimental (47.1% worsening). High pollution (37.0%) and high humidity (36.1%) were ranked as major aggravators. Low physical activity detrimentally affected 27.7%.

Spearman correlations confirmed perception–behavior concordance:

- Summer improvement ↔ beneficial high-sun perception: ρ = 0.444, *p* < 0.001.
- Winter worsening ↔ detrimental low-sun perception: ρ = 0.312, *p* < 0.001.

High pollution (37.0%) and high humidity (36.1%) were ranked as major aggravators. Interestingly, in contrast to sunlight, both high and low extremes of humidity were perceived primarily as factors leading to symptoms worsening (median score of 3 for both conditions). Low physical activity detrimentally affected 27.7%.

The perceived impact of physical activity demonstrated a distinct pattern. High physical activity was generally viewed as a neutral factor, with 56.3% (*n* = 67) of respondents reporting no change in symptoms (median score 4, *p* = 0.130). In contrast, low physical activity was identified as a clear aggravator; 27.7% (*n* = 33) of participants reported that a lack of exercise led to symptom worsening, resulting in a significantly lower median score of 3 (*p* < 0.001).

### 3.5. Ambient Antipsoriatic Radiant Exposure and Seasonal Psoriasis Course

To evaluate the independent association between ultraviolet (UV) exposure and seasonal psoriasis dynamics, mixed-effects logistic regression models were fitted using two clinically meaningful binary outcomes: (1) seasonal improvement (Likert score 5–7) and (2) seasonal worsening (Likert score 1–3). All four seasonal observations per participant were included (*n* = 476), and random intercepts for participant ID were incorporated to account for within-subject clustering across seasons. UV exposure was operationalized using predefined metric MED-adjusted declared exposure (UV_decl_/MED).

Each metric was categorized into quartiles (Q1–Q4), with the lowest quartile serving as reference. All models were adjusted for physical activity (any vs. none), vitamin D supplementation, BMI (continuous), and lesion exposure pattern (exposed vs. covered). Season was not entered as a covariate in quartile models because UV distribution was season-dependent by design; thus, these models estimate the overall exposure–response relationship across all seasonal observations.

#### 3.5.1. Ultraviolet Exposure Distribution

**Figure 2:**
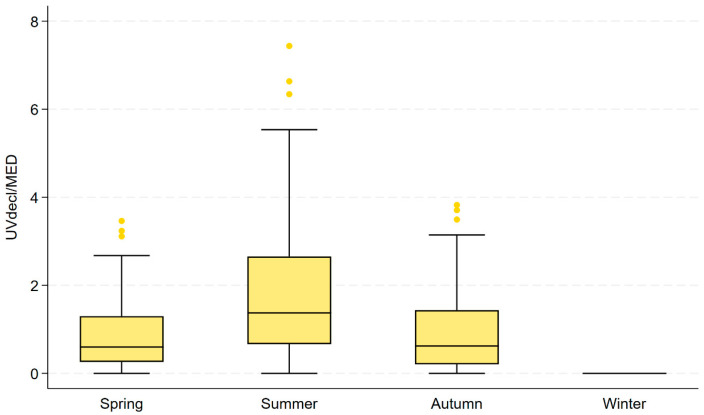
Seasonal distribution of UV_decl_/MED (boxplot by season).

Seasonal differences in MED-adjusted mean of declared daily ARE for spring, summer, and autumn were pronounced. Median UV_decl_/MED was highest in summer (1.372), compared with spring (0.598) and autumn (0.622), while winter exposure remained uniformly zero (Table 4). The distribution was right-skewed, particularly in summer, where the 90th percentile reached 4.52 and the maximum value 7.44, indicating substantial interindividual variability at higher exposure levels (Figure 2).

#### 3.5.2. Association Between UV Exposure and Seasonal Improvement

In mixed-effects logistic regression models including random intercepts for participant ID, higher quartiles of UV_decl_/MED were independently associated with increased odds of seasonal improvement (Table 5).

Compared with the lowest quartile (Q1), the odds ratios (OR) for improvement were:

- Q2: OR 3.42 (95% CI 1.60–7.34), *p* = 0.002.
- Q3: OR 5.34 (95% CI 2.50–11.43), *p* < 0.001.
- Q4: OR 4.65 (95% CI 2.04–10.58), *p* < 0.001.

These findings demonstrate a robust exposure–response relationship between increasing ARE and higher probability of seasonal improvement.

Random intercept variance (ID): 1.07. LR test vs. logistic model: *p* = 0.0005. Adjusted for physical activity, vitamin D supplementation, BMI, and lesion exposure pattern.

#### 3.5.3. Association Between UV Exposure and Seasonal Worsening

In the corresponding mixed-effects model for seasonal worsening, increasing UV exposure was associated with progressively lower odds of worsening. Compared with Q1:

- Q2: OR 0.29 (95% CI 0.15–0.55), *p* < 0.001.
- Q3: OR 0.21 (95% CI 0.11–0.40), *p* < 0.001.
- Q4: OR 0.16 (95% CI 0.08–0.33), *p* < 0.001.

The inverse association was consistent across all exposure levels and remained significant after adjustment for the same covariates (Table 6).

Exposure variable: UV_decl_/MED quartiles (reference = Q1). Random intercept variance (ID): 1.11 LR test vs. logistic model: *p* < 0.001. Adjusted for physical activity, vitamin D supplementation, BMI, and lesion exposure pattern.

#### 3.5.4. Continuous and Non-Linear Exposure Modeling

To further evaluate dose–response patterns, UV_decl_/MED was analyzed as a continuous variable. In continuous modeling, each one-unit increase in UV_decl_/MED was associated with:

- 1.73-fold higher odds of improvement (OR 1.73, 95% CI 1.32–2.27, *p* < 0.001).
- 0.53-fold lower odds of worsening (OR 0.53, 95% CI 0.42–0.67, *p* < 0.001).

The quadratic term was introduced into the mixed-effects models to assess potential non-linear effects. Because odds ratios (OR) are dynamic and non-constant in quadratic modeling, the effect estimates for these specific terms are presented as log-odds regression coefficients (β). For improvement, the positive linear coefficient (log-odds β = 0.885, 95% CI: 0.319–1.451, *p* = 0.002) reflects an initial dose-dependent increase in the probability of improvement. However, the negative quadratic coefficient (log-odds β = −0.115, 95% CI: −0.227 to −0.003, *p* = 0.044) acts as a mathematical modifier that attenuates this benefit at higher doses, curving the response downward. This combination mathematically defines a statistically significant inverted U-shaped relationship. The estimated turning point for the declared daily ARE was 3.85 MED (95% CI: 1.88–5.82, *p* < 0.001), suggesting attenuation of benefit at very high exposure levels (Figure 1A).

For worsening: Linear term: log-odds β = −0.872 (95% CI: −1.410 to −0.334, *p* = 0.002). Quadratic term: log-odds β = 0.064 (95% CI: −0.053 to 0.181, *p* = 0.281).

**Figure 3:**
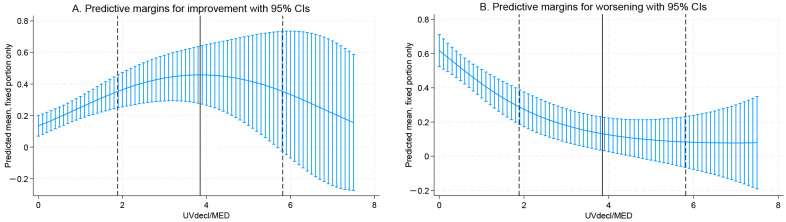
Non-linear relationship between modeled ambient ARE and the probability of seasonal psoriasis improvement. The curve represents the predicted probability of clinical improvement derived from a mixed-effects logistic regression model adjusted for physical activity, vitamin D supplementation, BMI, and skin exposure. The inverted U-shaped trend suggests a therapeutic window, with benefit diminishing at higher cumulative UV doses. Abbreviations: MED, Minimal Erythema Dose; UV, Ultraviolet; ARE, Antipsoriatic Radiant Exposure.

Since the quadratic term was not statistically significant, worsening demonstrated a predominantly linear protective relationship with increasing UV exposure (Figure 3B).

To ensure the robustness of the inverted U-shaped relationship, several sensitivity analyses were conducted. First, an ordinal mixed-effects model utilizing the full 7-point scale confirmed that the non-linear exposure–response shape persisted (*p* = 0.038), indicating it was not an artifact of dichotomizing the outcomes. Second, a stratified analysis excluding winter observations (where UV_decl_/MED is uniformly zero) maintained a significant positive association between UV exposure and seasonal improvement (*p* = 0.031), demonstrating that the UV metric is not merely acting as a proxy for season. Finally, excluding extreme exposure values (the top 5% of UV_decl_/MED observations) did not eliminate the statistical significance of the quadratic term (*p* = 0.004), confirming that the turning point is not solely driven by a small number of high-exposure outliers.

#### 3.5.5. Sensitivity Analysis Including Shift Work

Additional adjustment for night shift work did not materially alter the magnitude or direction of the UV effect estimates. In the fully adjusted quadratic mixed-effects model, MED-adjusted declared UV exposure remained independently associated with seasonal improvement (*p* = 0.002), and the quadratic term retained statistical significance (*p* = 0.044), supporting a non-linear exposure–response relationship. For seasonal worsening, the linear UV effect remained significant (*p* = 0.002), whereas the quadratic component was not statistically significant. Night shiftwork itself was not independently associated with seasonal improvement (*p* = 0.49) and demonstrated only a borderline association with seasonal worsening (*p* = 0.053).

Finally, to address potential confounding by clinical treatment patterns, an additional sensitivity analysis was performed incorporating patient history of clinical phototherapy as a covariate. In this fully adjusted mixed-effects model, the non-linear, inverted U-shaped exposure–response relationship between UV_decl_/MED and seasonal improvement remained robust and statistically significant (quadratic term log-odds β = −0.115, *p* = 0.045). This confirms that ambient UV exposure exerts an independent modulatory effect regardless of prior phototherapy interventions.

These findings confirm that the UV–psoriasis relationship is independent of occupational light exposure patterns.

## 4. Discussion

Seasonal variability in psoriasis activity is a well-recognized phenomenon that is typically characterized by winter exacerbations and summer improvements, although the exact magnitude varies across different geographical regions [25,26,27]. Our study confirmed a highly prevalent patient-perceived seasonality, reported by 89.9% of participants, which aligns with and even exceeds large-scale epidemiological observations [28]. For instance, a worldwide survey by Ferguson et al. [7] demonstrated that 77% of patients experienced seasonal changes, mostly worsening during the winter season. This pattern is supported by digital epidemiology, where global internet searches for psoriasis consistently peak in late winter and drop in late summer [9,10]. Moreover, objective clinical measurements using the Physician Global Assessment (PGA) confirm that psoriasis flares significantly during winter months [29]. Additionally, seasonal changes influence therapeutic management, with systemic biologic drug initiation peaking in spring following winter disease flares [11]. Despite this general trend, some studies, such as those from Japan, report no distinct seasonal frequency in healthcare utilization, and a systematic review from Northern Europe noted that only 30% of patients improved in summer [6,15]. This heterogeneity was also reflected in Chinese and Polish cohorts, highlighting that seasonality is a complex trait influenced by lifestyle and individual predispositions [8,12]. Furthermore, Mrowietz et al. [30] classified patients into distinct seasonal phenotypes, underlining that not all patients follow the classical winter-flare pattern.

The primary biological mediator of summer improvement is UV radiation [31,32]. In our multivariable mixed-effects models, higher MED-adjusted daily ARE was independently associated with significantly increased odds of seasonal improvement. The biological plausibility of this is well established; UVB exerts localized immunosuppressive effects, inducing apoptosis in hyperproliferating keratinocytes and pathogenic epidermal T-cells [33,34]. UV radiation also activates apoptotic, circadian, and redox signaling pathways in the epidermis, effectively suppressing the IL-23/Th17 axis which is central to psoriasis pathogenesis [35]. Furthermore, UV exposure promotes epigenetic alterations, such as the reversal of DNA methylation patterns in psoriatic lesions, which directly correlates with clinical clearance [36].

However, the most crucial finding of our study is the demonstration of an inverted U-shaped, non-linear relationship between continuous MED-adjusted declared daily ARE and the probability of seasonal improvement. Quadratic modeling estimated a distinct turning point at UV_decl_/MED = 3.85, beyond which the marginal clinical benefit attenuated. These findings indicate that cumulative UV dose does not translate into indefinitely linear therapeutic gain. Several pathological and adaptive mechanisms can explain this attenuation. First, prolonged UV exposure triggers photoadaptation, thickening the stratum corneum and progressively limiting further UV penetration [37]. Importantly, the relatively high proportion of self-reported phototypes IV–V in our cohort likely reflects self-classification bias and perceived tanning ability rather than true constitutional pigmentation distribution. Second, while UV is essential for synthesizing vitamin D, a hormone that regulates keratinocyte proliferation, excessive exposure leads to metabolic saturation and the formation of inactive metabolites like lumisterol, preventing further therapeutic gains [38,39]. Third, extreme UV exposure causing severe erythema acts as a physical trauma, potentially provoking the isomorphic Koebner response and triggering new plaques [40]. Finally, a specific subset of patients may experience photo-aggravation, where UV-induced reactive oxygen species create a pro-inflammatory microenvironment that overrides the desired immunosuppression [41].

While UV radiation plays a dominant role, psoriasis seasonality is heavily influenced by broader environmental and behavioral factors [19,42]. During winter, low ambient temperature and humidity significantly compromise epidermal barrier function [43]. Low humidity increases transepidermal water loss (TEWL), stimulating epidermal DNA synthesis and amplifying hyperproliferative inflammatory responses [44,45]. Winter air pollution also plays a critical pathogenic role [46]. Airborne pollutants, particularly particulate matter (PM_2.5_, PM_10_) and heavy metals like cadmium, directly activate the aryl hydrocarbon receptor (AhR), a transcription factor that enhances Th17 differentiation and exacerbates cutaneous inflammation [47,48]. Smoking, another major source of pollutants and AhR activation, interacts with cold weather to further aggravate the disease, as adjusted for in our models [49].

It is important to note that our findings regarding environmental triggers, such as humidity and air pollution, are based on patient perception rather than objective exposure measurements. For instance, the observation that both high and low extremes of humidity are perceived as aggravators may reflect general physical discomfort rather than a direct causal relationship. Similarly, reports that high pollution worsens symptoms could represent subjective symptom attribution. While these patient-perceived triggers provide valuable insights for individualized clinical counseling and hypothesis generation, objective environmental monitoring in prospective cohorts is strictly required to establish definitive causality [46,48,50,51].Emerging evidence also points to the role of chronobiology and circadian rhythms in the natural history of psoriasis [52]. Central and peripheral circadian clocks regulate skin homeostasis, and their disruption via shift work or wintertime darkness leads to systemic circadian misalignment [53]. This misalignment increases systemic inflammation and alters the diurnal expression of the IL-23 receptor, further contributing to winter flares and sleep disturbances [54].

### Study Limitations and Future Directions

The identification of the 3.85 UV_decl_MED threshold is strictly exploratory and hypothesis-generating. Values exceeding this turning point were observed exclusively during summer and accounted for 16.0% of summer observations (19/119), corresponding to 4.0% of all seasonal observations. Because this estimate depends on multiple methodological assumptions, including self-reported exposure time, self-assessed Fitzpatrick phototype, behavioral inference, and modeled ambient ARE data, it should not be framed as a clinically actionable recommendation. While it suggests an association where perceived benefits attenuate at extreme doses, external prospective validation is required before proposing any firm clinical counseling cut-offs. Although our models controlled several potential confounders, our study is not without limitations.

First, our pragmatic digital recruitment strategy via patient organizations and social media introduces potential selection bias. Patients engaging in online health communities are typically more health-literate and attuned to symptom tracking than the broader psoriasis population. This methodological enrichment likely attracted individuals already aware of their seasonal disease fluctuations, which may have inflated our seasonality prevalence estimate (89.9%) compared to unselected, population-based cohorts. The implications of this recruitment strategy significantly limit the generalizability of our findings. Our cohort’s responses may not accurately represent the broader, less symptomatic, or less engaged psoriasis population, and conclusions regarding exposure–response patterns must be interpreted with caution.

Second, our UV exposure metric relies on self-reported elements (phototype and outdoor time) and methodological assumptions regarding MED assignment. Self-reported skin phototype is frequently misclassified by patients, and our outdoor time metric does not capture whether the skin was exposed, the use of sunscreen, or specific shade-seeking behaviors. This introduces non-differential measurement error, which typically biases linear associations toward the null but can also distort the shape of non-linear exposure–response curves [55,56]. Biological variance in UV sensitivity can differ significantly even within the same phototype category, and a larger sample size would be required to mitigate the impact of such individual outliers on the calculated UV_decl_/MED metric. Consequently, our UV_decl_/MED index must be interpreted strictly as a proxy for biologically effective exposure rather than a direct dose measurement.

Third, several key confounders remain unmeasured, including specific systemic treatment types (e.g., biologics), travel to sunny climates, and tanning bed use. While we adjusted phototherapy history and shift work, the absence of real-time clinical scores (PASI/PGA) limits absolute causal interpretation. Furthermore, the study’s generalizability is limited by Poland’s narrow latitude band and predominantly fair constitutional phototypes. The observed overrepresentation of phototypes IV–V likely reflects self-classification bias regarding tanning ability rather than true demographic distribution.

Furthermore, ambient UV availability is intrinsically coupled with the season. Our behavioral exposure metric (MED-adjusted declared daily ARE) attempts to mitigate this by accounting for individual outdoor time—meaning that even during summer, individual exposure can be zero if a patient remains indoors. Additionally, our sensitivity analyses maintained significant exposure–response associations even when winter observations were excluded. Nevertheless, it remains difficult to fully disentangle the independent effect of UV from broader seasonal variations (e.g., changes in temperature, indoor heating, stress, or lifestyle). Consequently, the observed associations may partially reflect residual seasonal confounding rather than isolated UV effects.

Clinical counseling regarding sun exposure in psoriasis must be carefully balanced with the established risks of skin cancer and photoaging [57,58,59]. While our findings suggest a therapeutic window for moderate ambient UV exposure, patients should be advised on rigorous sun safety practices. This includes the strategic use of broad-spectrum sunscreens, which reduce the risk of painful erythema (a known trigger for the Koebner phenomenon) without eliminating the biological immunomodulatory effects of UV radiation. Furthermore, for patients requiring higher or more consistent UV doses, supervised narrow-band UVB (NB-UVB) phototherapy remains the gold standard. Unlike natural sunlight, clinical phototherapy offers precise dose control and a superior safety profile, making it the preferred option for achieving therapeutic benefits while minimizing long-term actinic damage, especially in patients treated with systemic immunosuppressants.

As dermatology increasingly moves toward AI-assisted clinical practice, the implications of this study extend beyond explaining psoriasis seasonality and into how environmental response patterns may eventually be monitored in routine care. Image-recognizing artificial intelligence models have shown very strong evidence in disease detection and inflammatory dermatologic image interpretation [60,61]. Because plaque psoriasis is a visually dynamic disease, with fluctuations in erythema, scale, thickness, and body-surface distribution across seasons, image-recognizing AI could help standardize the longitudinal assessment of disease severity. These systems can detect seasonal changes that may be difficult for patients or clinicians to quantify consistently [62]. In the context of this work, such tools could eventually be integrated with environmental and geospatial data, such as our MED-adjusted UV exposure metric, to create personalized monitoring models connecting visible skin changes with real-world exposure patterns. Ultimately, this would support more individualized clinical counseling regarding sun exposure and optimal treatment timing.

## 5. Conclusions

Patient-perceived seasonality of plaque psoriasis is highly prevalent and demonstrates a consistent directional pattern, characterized by improvement during summer and worsening during winter. This seasonal asymmetry is robust at the within-individual level and is not attributable to random variation. This study provides one of the first patient-level analyses integrating individualized UV exposure with perceived psoriasis seasonality. Objectively quantified ambient UV exposure, particularly when adjusted for minimal erythema dose, appears to partially explain the favorable summer course, supporting a biologically plausible photomodulatory effect. However, UV exposure alone does not fully account for seasonal variability, and its influence is limited outside the high-exposure months.

Behavioral factors, especially physical activity, modify seasonal disease expression, suggesting that lifestyle-related exposures may interact with environmental determinants. In contrast, vitamin D supplementation, BMI, lesion localization, phototype grouping, and shift work were not independently associated with seasonal course after accounting for within-subject clustering.

In conclusion, due to the cross-sectional, retrospective, and perception-based nature of this study, as well as selection bias introduced by online recruitment, these findings demonstrate epidemiological associations rather than causal effects. The high prevalence of seasonality and the observed inverted U-shaped relationship with ambient ARE, including the 3.85 MED turning point, are exploratory, hypothesis-generating observations. They highlight the complex structural coupling between season and UV exposure. Our findings should not be translated into clinical therapeutic recommendations without rigorous external validation in prospective, objectively measured clinical trials.

## Figures and Tables

**Table 1:** Population characteristics.

Characteristic	Value
Demographics	
Age, years, mean ± SD (range)	43.5 ± 12.5 (18–71)
Female sex, *n* (%)	82 (68.9)
BMI, kg/m^2^, mean ± SD	27.2 ± 4.9
Any comorbidity, *n* (%)	62 (52.1)
Shift work (any night), *n* (%)	29 (24.4)
Any physical activity, *n* (%)	97 (81.5)
Outdoor exposure (time outdoors per day)	
<2 h, *n* (%)	58 (48.7)
2–4 h, *n* (%)	33 (27.7)
≥4 h, *n* (%)	28 (23.5)
Self-reported skin phototypes	
Type I, *n* (%)	5 (4.2)
Type II, *n* (%)	19 (16.0)
Type III, *n* (%)	48 (40.3)
Type IV, *n* (%)	36 (30.3)
Type V, *n* (%)	11 (9.2)
Psoriasis lesion locations (multiple choice)	
Scalp, *n* (%)	78 (65.5)
Elbows, *n* (%)	92 (77.3)
Knees, *n* (%)	89 (74.8)
Lower legs (shins), *n* (%)	76 (63.9)
Nails, *n* (%)	45 (37.8)
Face, *n* (%)	32 (26.9)
Genitals, *n* (%)	28 (23.5)
Hands, *n* (%)	41 (34.5)
Feet, *n* (%)	35 (29.4)
Trunk (chest/back/abdomen), *n* (%)	67 (56.3)
Arms (upper), *n* (%)	54 (45.4)
Inverse (axillae/groin), *n* (%)	19 (16.0)
UV exposure	
Individual ambient ARE, J_psor_/m^2^, mean ± SD	494 ± 440

Baseline demographic and clinical characteristics of the study population (N = 119). Data are presented as mean ± standard deviation (SD) for continuous variables or as number (percentage) for categorical variables. Skin phototypes are reported based on participant self-assessment. Abbreviations: BMI, Body Mass Index; MED, Minimal Erythema Dose; SD, Standard Deviation.

**Table 2:** Participant-reported seasonal fluctuations in psoriasis course.

Season	Strong Worsening (1)	Moderate Worsening (2)	Mild Worsening (3)	No Change (4)	Mild Improvement (5)	Moderate Improvement (6)	Strong Improvement (7)
Spring	6 (5.0%)	14 (11.8%)	25 (21.0%)	39 (32.8%)	23 (19.3%)	8 (6.7%)	4 (3.4%)
Summer	5 (4.2%)	8 (6.7%)	6 (5.0%)	34 (28.6%)	28 (23.5%)	19 (16.0%)	19 (16.0%)
Autumn	16 (13.4%)	24 (20.2%)	35 (29.4%)	31 (26.1%)	7 (5.9%)	4 (3.4%)	2 (1.7%)
Winter	32 (26.9%)	29 (24.4%)	24 (20.2%)	22 (18.5%)	4 (3.4%)	5 (4.2%)	3 (2.5%)

Values represent scores on a 7-point Likert scale (1: marked worsening, 4: no change, 7: marked improvement). Statistical significance was determined using the Wilcoxon signed-rank test comparing observed scores to the neutral value of 4. Statistical analysis: Wilcoxon signed-rank test versus neutral score (=4): Spring *p* = 0.197; Summer *p* < 0.001; Autumn *p* < 0.001; Winter *p* < 0.001.

**Table 3:** Participant-reported environmental triggers in psoriasis course.

Exposure Factor	Worsening (1–3), *n* (%)	Neutral (4), *n* (%)	Improvement (5–7), *n* (%)	Median	*p* vs. Neutral
High sunlight	20 (16.8)	29 (24.4)	70 (58.8)	5	<0.001
Low sunlight	56 (47.1)	50 (42.0)	13 (10.9)	3	<0.001
High humidity	43 (36.1)	65 (54.6)	11 (9.2)	3	<0.001
Low humidity	33 (27.7)	68 (57.1)	18 (15.1)	3	0.025
High air pollution	44 (37.0)	70 (58.8)	5 (4.2)	3	<0.001
Low air pollution	17 (14.3)	82 (68.9)	20 (16.8)	4	0.657
High physical activity	31 (26.1)	67 (56.3)	21 (17.6)	4	0.130
Low physical activity	33 (27.7)	78 (65.5)	8 (6.7)	3	<0.001

Values represent scores on a 7-point Likert scale (1: marked worsening, 4: no change, 7: marked improvement). Statistical significance was determined using the Wilcoxon signed-rank test comparing observed scores to the neutral value of 4.

**Table 4:** Distribution of MED-adjusted declared UV exposure (UV_decl_/MED) by season.

Season	N	Mean	SD	Min	p10	p25	Median	p75	p90	Max
Spring	119	0.883	0.801	0.000	0.077	0.259	0.598	1.298	2.167	3.462
Summer	119	1.878	1.615	0.000	0.237	0.664	1.372	2.655	4.517	7.436
Autumn	119	0.948	0.928	0.000	0.048	0.207	0.622	1.437	2.451	3.824
Winter	119	0.000	0.000	0.000	0.000	0.000	0.000	0.000	0.000	0.000
Total	476	0.927	1.210	0.000	0.000	0.000	0.504	1.382	2.480	7.436

Abbreviations: MED, Minimal Erythema Dose; SD, Standard Deviation; p10–p90, 10th to 90th percentiles; UV_decl_/MED, individualized behaviorally weighted ambient ARE normalized to MED. Notes: Winter exposure values were uniformly zero due to negligible ambient UV irradiance and corresponding behavioral patterns (heavy clothing) limiting skin exposure during this season.

**Table 5:** Mixed-Effects Logistic Regression Model for Seasonal Improvement (Quartile Model).

Variable	OR	95% CI	*p*-Value
UV_decl_/MED quartiles			
Q2 vs. Q1	3.42	1.60–7.34	0.002
Q3 vs. Q1	5.34	2.50–11.43	<0.001
Q4 vs. Q1	4.65	2.04–10.58	<0.001
Any physical activity	0.75	0.33–1.66	0.473
Vitamin D supplementation	1.09	0.56–2.14	0.800
BMI (per 1 kg/m^2^)	1.02	0.96–1.09	0.507
Exposed lesions	1.08	0.50–2.30	0.851

**Abbreviations:** BMI, Body Mass Index; CI, Confidence Interval; MED, Minimal Erythema Dose; OR, Odds Ratio; Q, Quartile; Ref., Reference category. Notes: Results were derived from a multivariable mixed-effects logistic regression model, incorporating random intercepts for participant ID to account for the non-independence of repeated seasonal observations. The model estimates the independent odds of patient-reported seasonal improvement. All listed covariates (physical activity, vitamin D supplementation, BMI, and lesion localization) were mutually adjusted in the model. A likelihood-ratio (LR) test confirmed that the mixed-effects model provided a significantly better fit than standard logistic regression (*p* < 0.001).

**Table 6:** Mixed-Effects Logistic Regression Model for Seasonal Worsening (Quartile Model).

Variable	OR	95% CI	*p*-Value
UV_decl_/MED quartiles			
Q2 vs. Q1	0.29	0.15–0.55	<0.001
Q3 vs. Q1	0.21	0.11–0.40	<0.001
Q4 vs. Q1	0.16	0.08–0.33	<0.001
Any physical activity	0.55	0.25–1.19	0.127
Vitamin D supplementation	1.17	0.62–2.22	0.623
BMI (per 1 kg/m^2^)	0.96	0.90–1.02	0.147
Exposed lesions	1.61	0.78–3.33	0.195

**Abbreviations:** BMI, Body Mass Index; CI, Confidence Interval; MED, Minimal Erythema Dose; OR, Odds Ratio; Q, Quartile; Ref., Reference category. **Notes:** Results were derived from a multivariable mixed-effects logistic regression model, incorporating random intercepts for participant ID to account for the non-independence of repeated seasonal observations. The model estimates the independent odds of patient-reported seasonal worsening. All listed covariates (physical activity, vitamin D supplementation, BMI, and lesion localization) were mutually adjusted in the model. A likelihood-ratio (LR) test confirmed that the mixed-effects model provided a significantly better fit than standard logistic regression (*p* < 0.001).

**Figure A1:**
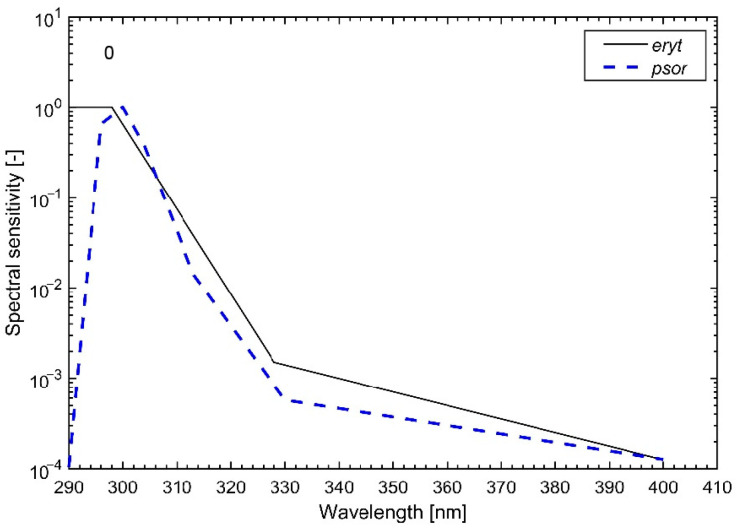
Normalized action spectra for the specific biological effects: erythema appearance (solid black, according to ISO/CIE, 2019) and psoriasis clearance (dashed blue, according to Krzyścin et al. [32]).

## Data Availability

The raw data supporting the conclusions of this article will be made available by the authors on request.

## Abbreviations

The following abbreviations are used in this manuscript:

AhR	Aryl Hydrocarbon Receptor
ARE	Antipsoriatic Radiant Exposure
BMI	Body Mass Index
CI	Confidence Interval
ID	Identifier (Participant ID)
IL	Interleukin (e.g., IL-17, IL-22, IL-23)
J_eryt_, J_psor_	Joules weighted according erythemal and antipsoriatic action spectrum, respectively
MED	Minimal Erythema Dose
OR	Odds Ratio
PASI	Psoriasis Area and Severity Index
PGA	Physician Global Assessment
PM	Particulate Matter (e.g., PM_2.5_, PM_10_)
Q	Quartile (e.g., Q1–Q4)
TEWL	Transepidermal Water Loss
Th17	T helper 17 cells
UV	Ultraviolet

## Appendix A

Antipsoriatic radiant exposure (ARE) from the definition is calculated as an integration of irradiance over spectral range, weighted with antipsoriatic action spectrum [32], and then over period of time. In the presented research, the antipsoriatic irradiance is estimated from erythemally weighted irradiance (ISO/CIE 2019), using previously derived by Institute of Geophysics, Polish Academy of Sciences model [63]. Erythemally weighted irradiance for the clear-sky is calculated with Allaart model [64]. The first step was to calculate erythemal and antipsoriatic irradiances (in W/m^2^) for the period from 6:00 to 18:00 (CET), with 1 h resolution, starting from 6:30 and ending at 17:30, for each day from April to September, from 2021 to 2025, for the clear-sky conditions. Then, the antipsoriatic irradiance was transformed to 1 h ARE (in J_psor_/m^2^) for all-sky conditions, using the data from CAMS (Copernicus Atmosphere Monitoring Service) solar radiation time-series [65] and the model to calculate cloud modification factor [66]. An average values for spring (April), summer (June and July) and autumn (September) were calculated across 5 years as 1 h sums for the period from 6:00 to 18:00 (CET), but also for the periods declared by the patients in the survey, i.e., 6:00–8:00, 8:00–10:00, 10:00–14:00, 14:00–16:00, 16:00–18:00. All ambient ARE means for each period and season were calculated at the center of grid cells corresponding to patients’ residential locations. Thus, total ARE is the mean ambient ARE accumulated between 06:00 and 18:00 assigned for each patient in each grid. Declared UV (behavioral exposure) is an ambient ARE summed in the self-reported average daily time periods spent outdoors in each season (UV_decl_). For example, two people from one cluster on summer can have UV_decl_ equal to 467.25 J_psor_/m^2^ (time declared from 16:00 to 18:00) or 2080.1 J_psor_/m^2^ (time declared from 8:00 to 18:00). Assuming MED = 300 J_eryt_/m^2^, these values result in corresponding UV_decl_/MED values of 1.56 and 6.93 (well above the threshold of 3.85).
